# Progress in the study of pentraxin-3(PTX-3) as a biomarker for sepsis

**DOI:** 10.3389/fmed.2024.1398024

**Published:** 2024-07-03

**Authors:** Yi Zhang, Xuelin Li, Xiaobei Zhang, Tiantian Wang, Xiangcheng Zhang

**Affiliations:** Department of Critical Care Medicine, The Affiliated Huai’an No 1 People’s Hospital of Nanjing Medical University, Huai’an, Jiangsu, China

**Keywords:** sepsis, pentraxin-3 (PTX-3), biomarker, diagnosis, prognosis

## Abstract

Sepsis is a intricate pathological process characterized by life-threatening organ dysfunction resulting from a dysregulated host response to infection. It stands as a prominent cause of mortality among critically ill patients globally. The pivotal focus in sepsis management lies in the early identification and prompt administration of antimicrobial agents. Owing to the constraints of current diagnostic methodologies, marked by insufficient sensitivity and delayed outcomes, extensive research has been undertaken to ascertain novel biomarkers for sepsis. In this review, we provide an overview discussing the latest advancements in the study of PTX-3 as a biomarker for sepsis. We acknowledge pivotal discoveries from preceding research and engage in discourse regarding the challenges and limitations confronted by PTX-3 as a sepsis biomarker.

## 1 Introduction

Sepsis is characterized by life-threatening organ dysfunction resulting from a dysregulated host response to infection (1). Recent research estimates globally recorded sepsis cases at 489 million, with reported sepsis-related deaths reaching 11 million, accounting for 19.7% of global mortality (2). In China, a study indicated a higher burden of sepsis in hospitalized patients compared to estimates (3). Therefore, early diagnosis of sepsis is crucial, as it allows for the prompt initiation of supportive and antibiotic therapy to reduce sepsis mortality. However, due to the complexity and heterogeneity of sepsis, accurate early diagnosis and prognosis assessment are challenging. Traditional diagnostic methods are both time-consuming and expensive, and require operation by professionally medically trained personnel (4). Blood culture is the gold standard for diagnosing sepsis; however, it has a high false-negative rate and often results in delayed outcomes (5, 6). Over the past few decades, extensive research has explored various biomarkers, utilizing them to diagnose and predict outcomes in sepsis patients, playing crucial roles in the pathophysiology of sepsis.

Pentraxin-3 (PTX-3), also known as human serum penetratin-3, is a representative member of the long-chain pentraxin subfamily in the pentraxin family (7). PTX-3 is a new type of acute-phase inflammatory factor and plays an important role in infectious diseases. In recent years, it has been found that the PTX-3 level increases sharply in the blood of sepsis patients, and may be superior to traditional biomarkers in judging the severity of their condition and prognosis assessment. The purpose of this review is to summarize the current literature on one of the sepsis biomarkers, PTX-3, to provide a comprehensive overview of the research progress on PTX-3 as a sepsis biomarker. This aims to offer a brief outlook for further research on the sepsis biomarker PTX-3.

## 2 Pentraxin-3(PTX-3)

Pentraxins is an evolutionarily conserved protein superfamily characterized by a structural motif, that is, the pentraxin domain (8). Pentraxin-3 is a 45-kilodalton protein that forms high-molecular-weight oligomers through interchain disulfide bonds (9). The C-terminal domain (203 amino acids) of PTX-3 has homology with classic short pentameric proteins such as C-reactive protein (CRP) and serum amyloid protein P (SAP), while its N-terminal domain (178 amino acids) has no significant homology with other known proteins.

Unlike classic short pentameric proteins such as CRP and SAP that are systemically produced by hepatocytes, PTX-3 is produced by various cell types and shows differences in genomic organization, cellular source, and ligand-binding characteristics (8). Due to significant differences in sequence and regulation, CRP (which is not an acute-phase protein in mice) cannot be used in genetic methods to study its *in vivo* functions, while PTX-3 remains highly conserved throughout the evolutionary process. Notably, PTX-3 plays a complex and irreplaceable role *in vivo*, being able to recognize various pathogens, regulate complement activity by binding to C1q, and promote the recognition of pathogens by macrophages and dendritic cells. PTX-3, as a liquid-phase effector molecule of the innate immune system, its production is stimulated by cytokines and bacterial endotoxins. PTX-3 can bind to specific pathogens, activate complement, promote cell recognition and clearance, and can also act as a matrix component (8).

PTX-3 is an acute-phase reactant with relatively low blood levels under normal circumstances (about 25 ng/mL in mice and < 2 ng/mL in humans). It has been reported that human plasma PTX-3 levels are related to gender, significantly lower in men than in women, and increase significantly with age. In infectious shock, sepsis, and other inflammatory and infectious states, plasma PTX-3 levels increase sharply, reaching a peak level within 6–8 h (10), and its concentration can increase up to 100 ng/mL during sepsis (11). When injury occurs, the PTX-3 level in the blood is extremely high, which is related to the release of pre-formed PTX-3 in neutrophils. Maugeri et al. (12) clearly described this, reporting that neutrophils led to an increase in plasma PTX-3 concentration within 6 h after the appearance of clinical symptoms of acute myocardial infarction and returned to normal within 48 h. Studies using *in vivo* neutrophil depletion and multiple vascular proteomics have concluded that PTX-3 may be stored and released in a polymeric form. Proteomic analysis of the aorta of mice injected with lipopolysaccharide (LPS) showed that PTX-3 was the most upregulated protein, and polymeric PTX-3 was deposited along with other neutrophil-derived proteins as early as 2 h after LPS injection. In healthy volunteers, a rapid degranulation reaction was observed 6 h after LPS injection, followed by an acute-phase response (13). In addition, studies have shown that plasma PTX-3 levels increase as the glomerular filtration rate (GFR) decreases (14), and the increase in liver PTX-3 levels in liver necrosis may be a marker of acute histological liver injury (15).

PTX-3 has a tendency to rise faster in the pathogenesis of sepsis than the previously used biomarkers and is superior to traditional biomarkers, which may be due to the fact that it is locally produced at the site of infection or tissue damage rather than relying on other molecules to trigger the synthesis of body organs. In contrast, CRP is systemically generated by liver cells in response to IL-6 stimulation, and the process takes longer and only begins to change significantly after 24–30 h (16). The comparison of PTX-3 with other common biomarkers is shown in Table 1.

**Table 1:** Comparison of PTX-3 with other commonly used biomarkers in sepsis.

	PTX-3	CRP	PCT
Normal values	< 2.0 ng/mL	0.8 mg/dL	< 0.5 ng/mL
Source	Neutrophils and diverse cells	Liver	Macrophages and diverse cells
Time to increase after insult	2 h	4-6 h	3-4 h
Time to peak concentration	6-8 h	36-50 h	6-24 h
Half-life	NR	19 h	22-35 h
Chronic liver failure	Elevation	Slight decrease	No effect
Renal failure	Elevation	No effect	Elevation
Renal replacement therapy	NR	No effect	Decrease

PTX-3, Pentraxin-3; CRP, C-reactive protein; PCT, Procalcitonin; NR, Not Report.

The overexpression of PTX-3 will exacerbate the inflammatory response and reduce the survival rate of mice subjected to intestinal ischemia–reperfusion injury (17). The data collected from various pathological processes indicate that there is a correlation between plasma PTX-3 level and disease severity, suggesting the potential role of PTX-3 as a pathological biomarker. Whether the significant correlation between the result and the severity reflects its role in the pathogenesis of the injury mechanism, such as amplifying the complement and coagulation cascade reactions, remains to be elucidated (18). Elevated PTX-3 levels have been observed in various infectious diseases including sepsis, septic shock, aspergillus infection, tuberculosis and dengue fever (19, 20). PTX-3 is also expressed in aseptic inflammation. It has been reported that the PTX-3 level of patients with acute coronary syndrome increases by about 6–7 ng/mL (21, 22), the PTX-3 level of patients with congestive heart failure increases (about 3–4 ng/mL) (23), the PTX-3 level in renal failure increases by about 5–6 ng/mL (14, 24), and the PTX-3 level in acute respiratory distress syndrome increases by about 70 ng/mL (25). In addition, in patients with AMI with ST-segment elevation, PTX-3 can predict the 3-month mortality rate after adjusting important risk factors and other acute phase prognostic indicators (26). Early detection of PTX-3 level is an independent indicator for predicting multiple organ dysfunction syndrome (MODS) and premature death in patients with cardiac arrest (27, 28). In autoimmune diseases, PTX-3 mediates the complement regulatory mechanism, leading to inflammation and tissue damage in RA (29–31). PTX-3 may be a new non-invasive biomarker indicating the clinical arthritis activity of RA patients (32). In addition, PTX-3 is significantly correlated with the activity degree of SLE (33, 34). PTX-3 may participate in the pathogenesis of psoriasis and can indicate the disease activity of psoriasis (35, 36). The increase of PTX-3 level during the acute attack of acute rheumatic fever may help predict the clinical course (37). PTX-3 can also be used for the early severity assessment and prediction of acute pancreatitis (AP) (38). However, PTX-3 is not as good as CRP and APACHE II score in predicting the mortality of AP, and the combination of PTX-3 and CRP cannot improve the predictive value of CRP (39).

In conclusion, PTX-3 is a multifunctional protein at the intersection of immunity, inflammation, extracellular matrix construction, and female reproduction (8, 40). In all these cases, the PTX-3 level is correlated with the clinical activity of the disease, making it a candidate biomarker for disease monitoring (41).

## 3 Diagnostic value

As an acute-phase reactant protein, PTX-3 has been widely studied as a biomarker to distinguish common bacterial infections from sepsis or septic shock. The results of one study showed that the area under the curve (AUC) for the discrimination between the healthy control group and the SIRS group was 0.922 (cut-off value 16.0 ng/mL, sensitivity 89.1%, specificity 85%), and the difference was statistically significant (42). At the same time, among suspected infection patients visiting the emergency department, the AUC of PTX-3 in predicting severe sepsis from day 0 to day 28 was 0.73 (cut-off value 14.1 ng/mL, sensitivity 63%, specificity 80%) (43). Another study showed that in adult febrile patients in the emergency department, the AUC for predicting bloodstream infection was 0.71, the critical value was 16.1 ng/mL, and the sensitivity was 76% and the specificity was 61% (44). Hamed et al. (45) studied the PTX-3 level on the 1st, 3rd, and 8th days of treatment in ICU sepsis patients and found that at the cut-off value of 5 μg/L, the lowest sensitivity and specificity were 92% and 64%, respectively. Moreover, this study defined a unified diagnostic boundary, diagnosing sepsis at least (≥5.0 ng/mL) and septic shock (≥9.0 ng/mL) (45).

Similar to adults, PTX-3 can be used as a reliable biomarker for neonatal sepsis with high sensitivity and specificity. One study showed that when using the PTX-3 cut-off value of 5.6 μg/L to diagnose sepsis, the sensitivity was 98.3%, the specificity was 96.7%, the positive predictive value (PPV) was 98%, and the negative predictive value (NPV) was 96%. While the critical value of CRP was 8 mg/dL, the sensitivity was 96.7%, the specificity was 96.7%, the positive predictive value (PPV) = 98.3%, and the negative predictive value (NPV) = 93.5%, and the accuracy (area under the curve) = 0.989 (46). Although this study may have some limitations, for the diagnosis of neonatal sepsis, the sensitivity of biomarkers is more important than the specificity, so PTX-3 seems to be superior to CRP as a diagnostic biomarker for neonatal sepsis.

## 4 Prognostic value

Abnormally elevated plasma PTX-3 levels are closely related to the mortality rate of sepsis and have important significance in predicting the risk of death for sepsis patients. Multiple studies have shown that the systemic PTX-3 level of critically ill patients with a fatal outcome is significantly higher than that of surviving critically ill patients (25, 42, 47–50). Among sepsis patients, the maximum PTX-3 value of non-surviving patients in the first to fourth days is significantly higher than that of surviving patients (44.8 vs. 6.4 ng/mL, 𝑃<0.001), and the AUC for predicting the case fatality rate is 0.82 (cut-off value 15 ng/mL, sensitivity 72%, specificity 81%) (51). Similarly, when predicting the mortality rate on the 28th day, the AUC of suspected infected emergency patients is 0.69 (95% confidence interval 0.58 ~ 0.79, *p* < 0.001), and the critical value is 7.7 ng/mL, (sensitivity 70%, specificity 63%) (43). Wang et al.’s (52) meta-analysis included 17 studies with 3,658 sepsis patients, and the results showed that the PTX-3 level of sepsis patients who died was significantly higher than that of surviving patients, indicating that a high level of PTX-3 is significantly related to the risk of death in sepsis and can predict the patient mortality rate. Lee et al.’s (53) meta-analysis found that an increase in PTX-3 doubles the risk of death in sepsis patients. Another trial conducted a prospective study on 160 sepsis patients and showed that when predicting the 28-day mortality rate of sepsis, at the cut-off value of 26.90 ng/mL, its sensitivity is 88.9% and specificity is 49.5%, and the AUC is 0.734 (95% CI 0.656 ~ 0.811) (54). A prospective cohort study by Kim et al. found that compared with PCT, neutrophil count and CRP, PTX-3 has a larger AUC value (0.819, 95% CI 0.677 ~ 0.961) in predicting the 28-day all-cause mortality rate of severe sepsis patients. At the same time, by establishing a Cox proportional hazards model, it was found that the plasma PTX-3 level measured at admission is an independent predictor of the 28-day all-cause mortality rate of severe sepsis patients (HR = 7.16, 95% CI 2.46 ~ 15.85) (55).

The above research findings suggest that PTX-3 is an early biomarker for predicting the mortality rate of sepsis. Timely detection of PTX-3 can provide guidance for subsequent treatment and management, which has significant clinical importance. Additionally, a single-center prospective study reveals that in comparison to PCT, IL-6, CRP, lactic acid, and platelet count, PTX-3 demonstrates a higher diagnostic value in differentiating between patients with infectious shock and those without (*p* < 0.001) (56). However, when compared to the traditional Simplified Acute Physiological Score II (SAPS II), the ability of PTX-3 to predict mortality is poorer (49).

In different studies, the optimal cut-off value of PTX-3 for predicting bacterial infection, sepsis, septic shock and mortality varies, and the reported sensitivity and specificity are also discrepant. The comparison with other indicators is presented in Table 2.

**Table 2:** The cut-off values of PTX-3 and other commonly used indicators in differencing infection, sepsis and predicting mortality.

Parameter	Clinical relevance	Cut-off	AUC	Sensitivity (%)	Specificity (%)	References
PTX-3	Diagnosis of sepsis	5 ng/mL	0.92	98	79	Hamed et al. (45)
	Diagnosis of septic shock	9 ng/mL	0.81	93	45	Hamed et al. (45)
	Diagnosis of Sepsis	NR	0.73	NR	NR	Muller et al. (49)
	Diagnostic value of sepsis	5.84 ng/mL	0.68	0.667	0.697	Chen et al. (56)
	Diagnostic value of septic shock	11.12 ng/mL	0.73	0.555	0.828	Chen et al. (56)
	Prognostic value of ICU mortality in patients with sepsis	NR	0.63	NR	NR	Muller et al. (49)
	Predicting 28-day mortality in patients with sepsis	7.7 ng/mL	0.69	70	63	Rottman et al. (43)
	Predicting 28-day mortality in patients with sepsis	26.9 ng/mL	0.734	88.9	49.5	Song et al. (54)
	Prediction of bloodstream infection	7.96 ng/mL	NR	90	29	de Kruif et al. (44)
	Prediction of bloodstream infection	16.1 ng/mL	NR	76	61	de Kruif et al. (44)
CRP	Septic patients compared to control group	8.02 mg/L	0.98	88.4	100	Hou et al. (57)
	Differentiating infectious and non-infectious SIRS	50 mg/L	NR	98.5	75.0	Póvoa et al. (58)
	Prognostic value of ICU mortality in patients with sepsis	NR	0.55	NR	NR	Muller et al. (49)
PCT	Cut-off for sepsis	1.57 ng/mL	NR	67.33	65.79	Aksaray et al. (59)
	Differentiating infectious and non-infectious SIRS	1.1 ng/mL	NR	97	78	Harbarth et al. (60)
	Differentiating infectious and non-infectious SIRS	1.8 ng/mL	NR	95	88	Rau et al. (61)
	Diagnostic value of sepsis	1.62 ng/mL	0.79	0.815	0.697	Chen et al. (56)
	Diagnostic value of septic shock	7.27 ng/mL	0.73	0.328	0.672	Chen et al. (56)
	Predicting 28-day mortality in patients with sepsis	0.47 ng/mL	0.689	92.1	41.2	Song et al. (54)
Lactate	Diagnostic value of sepsis	2.3 mmol/L	0.70	0.723	0.621	Chen et al. (56)
	Diagnostic value of septic shock	3.9 mmol/L	0.73	0.453	0.914	Chen et al. (56)
SOFA	Predicting 28-day mortality in patients with sepsis.	8 points	0.712	81.0	51.5	Song et al. (54)
SAPS II	Prognostic value of ICU mortality	NR	0.76	NR	NR	Muller et al. (49)

PTX-3, Pentraxin-3; CRP, C-reactive protein; PCT, Procalcitonin; SOFA, Sequential Organ Failure Assessment; SAPS II, Simplified Acute Physiological Score II; AUC, Area under curve; NR, Not Report.

The correlation between the PTX-3 level and the disease severity and organ dysfunction exceeds that of other measured mediators such as tumor necrosis factor-α, interleukin-6, and C-reactive protein (62). Although PTX-3 alone may not perform as well as the Simplified Acute Physiology Score II (SAPS II), this does not necessarily rule out its potential application in prognosis. In recent decades, scoring systems such as the Acute Physiology and Chronic Health Evaluation System (APACHE II), the Simplified Acute Physiology Score (SAPS), and the Sequential Organ Failure Assessment (SOFA) have become increasingly popular in assessing the risk of death in critically ill patients. However, these scoring systems have significant limitations in clinical practice. Data collection requires multiple laboratory measurements and the calculation involving numerous variables, making it both laborious and costly (63, 64).

## 5 Discussion

Although PTX-3 has good prospects as a biomarker for sepsis, challenges and limitations still need to be addressed. Due to the heterogeneity of research and selection criteria, an accurate cut-off value has not been determined yet. Therefore, laboratory test results should be interpreted in combination with the clinical situation and used in combination with other laboratory findings to ensure accurate diagnosis and guide appropriate management. Previous studies have included studies involving both adults and children, as well as individuals with different severities in infectious diseases. Some scholars strongly recommend subgroup analysis according to age and different disease manifestations (65). Future research should focus on refining the role of PTX-3 in different etiologies of sepsis and evaluating its potential as a therapeutic target, such as whether the PTX-3 level can guide the use of antibiotics, etc., and its molecular biological mechanism still needs to be further clarified.

## Summary

PTX-3 appears to be a promising prognostic biomarker for critically ill patients. Currently, research is limited to observational designs estimating the predictive potential for mortality risk. Further investigation is needed to determine whether monitoring PTX-3 levels during treatment can be used to guide therapeutic decisions. In conclusion, PTX-3 emerges as a valuable candidate as a biomarker for sepsis, providing insights into its structural characteristics, physiological functions, and potential diagnostic applications. The progress in understanding the role of PTX-3 in sepsis paves the way for improving diagnostic accuracy, prognostic assessment, and targeted therapeutic interventions.

## Author contributions

YZ: Writing – original draft, Writing – review & editing, Conceptualization, Data curation, Formal analysis, Investigation, Methodology, Funding acquisition. XL: Conceptualization, Data curation, Formal analysis, Investigation, Methodology, Software, Writing – review & editing. XiaoZ: Conceptualization, Investigation, Methodology, Software, Writing – review & editing. TW: Conceptualization, Formal analysis, Investigation, Methodology, Project administration, Writing – review & editing. XianZ: Methodology, Supervision, Validation, Writing – review & editing.
